# Association between STAT3 gene Polymorphisms and Crohn’s disease susceptibility: a case–control study in a Chinese Han population

**DOI:** 10.1186/1746-1596-9-104

**Published:** 2014-05-29

**Authors:** Zhengting Wang, Bin Xu, Hongxin Zhang, Rong Fan, Jie Zhou, Jie Zhong

**Affiliations:** 1 Department of Gastroenterology, Ruijin Hospital, School of Medicine, Shanghai Jiaotong University, Shanghai 200025, China; 2 State Key Laboratory of Medical Genomics, Research Center for Experimental Medicine, Shanghai Institute of Hematology, Ruijin Hospital, Shanghai Jiaotong University School of Medicine, Shanghai 200025, China

**Keywords:** STAT3, Polymorphism, Crohn’s diseases, Susceptibility, Association study

## Abstract

**Background:**

Crohn’s disease (CD) is an immune-related disease with genetic predisposition. This study aimed to investigate the association of three polymorphisms in the signal transducer and activator of transcription 3 (STAT3) gene with CD risk in a Chinese population.

**Methods:**

We conducted a hospital-based case–control study involving 232 CD patients and 272 controls. Genotyping was performed using polymerase chain reaction with sequence-specific primer method. Statistical analyses were conducted using logistic regression and genotype risk scoring.

**Results:**

Significant differences were found between patients and controls in allele/genotype distributions of rs744166 (*P*allele = 0.0008; *P*genotype = 0.003) and allele distributions of rs4796793 (*P* = 0.03). The risk for CD associated with the rs744166-A mutant allele decreased by 37% [95% confidence interval (CI): 0.48–0.83] under the additive model, 39% (95% CI: 0.43–0.81) under the dominant model and 57% (95% CI: 0.24–0.77) under the recessive model. Carriers of the rs4796793-G mutant allele exhibited 25% (95% CI: 0.58–0.98; *P* = 0.03) and 47% (95% CI: 0.30–0.95) decreased risks of developing CD under the additive and recessive models, respectively.

**Conclusions:**

STAT3 rs744166 and rs4796793 polymorphisms may be associated with CD occurrence and used as a predictive factor of CD in Chinese Han populations.

**Virtual Slides:**

The virtual slide(s) for this article can be found here: http://diagnosticpathology.slidepath.com/webViewer.php?snapshotId=1297687014

## Background

Crohn’s disease (CD) and ulcerative colitis (UC) are inflammatory bowel diseases (IBDs). The etiology and pathogenesis of CD are not completely understood. However, familial aggregation and twin studies report that patients with CD carry strong genetic predisposition [1]. Several studies also strongly suggest that CD results from a combination of factors, such as commensal bacteria, food antigens, immunologic factors and multiple genetic factors [2,3]. The signal transducer and activator of transcription 3 (STAT3) gene is a potential candidate gene for CD for several reasons. STAT3 is a member of STAT family, which possesses an important function in the development of human immune system and haematopoiesis. This gene has been associated with the signal transduction pathway of multiple cytokines, including IL-2/γc, IL-6/gp130, IFN and IL-10 families, as well as IL-12, IL- 23, Flt3 ligand, M-CSF, G-CSF, leptin and growth hormone [4-9]. Several studies have highlighted that the STAT3 signaling pathway is important in the occurrence and development of IBD both in patients and animal models [10-13].

In 2008, Barrett et al. [14] reported that the STAT3 locus is significantly associated with CD susceptibility in a genome-wide association study (GWAS). Since then, a number of studies have demonstrated that the polymorphisms of STAT3 are associated with CD as well as UC, but their results are not consistent in different population cohorts [15-20]. Therefore, we performed an analysis on three polymorphisms (rs2293152, rs4796793 and rs744166) of STAT3 and CD in Chinese Han population.

## Methods

### Patient and control subjects

This hospital-based case–control study involved 232 CD patients and 272 healthy controls of Chinese Han population recruited from the Department of Gastroenterology of Ruijin Hospital, which is connected with the Shanghai Jiaotong University School of Medicine between January 2009 and December 2010. Senior physicians diagnosed all patients based on clinical, endoscopic, radiological and histopathological findings in accordance with previously established international criteria [21]. All patients were followed up at least for 1 year and registered with an integrated clinical and epidemiological registry. Controls were randomly selected from healthy persons under routine health screening. The present study was performed in accordance with the principles of Declaration of Helsinki and approved by the Research Ethics Committee of Ruijin Hospital, Shanghai, China. Informed consent was obtained from all subjects before blood sampling was carried out.

### Genotyping

Genomic DNA was isolated from Ethylene Diamine Tetraacetic Acid (EDTA) peripheral blood using the QIAamp blood extraction kit (Qiagen, Hilden, Germany) following the manufacturer’s instructions. All DNA samples were genotyped for single nucleotide polymorphisms by polymerase chain reaction with sequence-specific primers (PCR-SSPs). All primers for the PCR-SSPs were designed using the genomic sequences in GenBank (http://www.ncbi.nlm.nih.gov). The primer sequences are listed in Table 1. The amplified products were assessed for the presence/absence of PCR amplicons specific to particular alleles using a standard 2% agarose gel electrophoresis, followed by ethidium-bromide staining. About 10% of the samples were then confirmed by sequencing.

**Table 1:** The primer sequence used for genotyping

**rs2293152**	**Internal control forward primer**	**CCGTTTAACCTAACTTCAT**
	**Common reverse primer**	**CCAGTTGTCTTTCATCCC**
	**Specific primer C**	**ACAAAGGGCCTCTGGCTGCC**
	**Specific primer G**	**ACAAAGGGCCTCTGGCTGCG**
rs4796793	Internal control forward primer	TCTGGTAGACACAGCTCAGTATGG
	Common reverse primer	CCATAGTCGCAGAGGTAGATTTTA
	Specific primer C	TGTTTAGTGATTTACTGCTTACAAAGG
	Specific primer G	TGTTTAGTGATTTACTGCTTACAAAGC
	Internal control forward primer	TGCCTCTGCCTCTTTTCCTG
	Common reverse primer	GATGGGACTTGGTGACTGACTG
	Specific primer C	TGTCTTGAGGGAATCGAGCC
	Specific primer G	ATGTCTTGAGGGAATCGAGCT

### Statistical analysis

For continuous and categorical variables, unpaired *t*-test and *χ*^2^ were conducted to compare CD patients and controls, respectively. To avoid gross genotyping error, all polymorphisms were evaluated for consistency with Hardy–Weinberg equilibrium on a contingency table of observed-versus-predicted genotype frequencies by using Pearson *χ*^2^ test or Fisher's exact test. Genotypes were compared by logistic regression analysis under assumptions of additive, dominant and recessive models of inheritance. A *P* < 0.05 was considered statistically significant.

## Results

Table 2 shows detailed information of patients and controls. Cases and controls were well matched by age and gender distribution.

**Table 2:** Characteristics of CD patients and healthy controls in the Chinese Han population

**Characteristics**	**CD patients**	**Control subjects**
Number	232	272
Age, mean ± SD (years)	33.6 ± 13.5	46.4 ± 9.8
Age range (years)	20-70	18-70
Male /female	149/83	172/100
Smoking (%)	53 (22.84)	67 (24.63)
Drinking (%)	32 (13.79)	39 (14.34)
Appendectomy (%)	15 (6.47)	18 (6.62)
Family history of CD	0	0

The frequencies and distributions of alleles and genotypes at rs2293152, rs4796793 and rs744166 STAT3 were identified and compared between CD patients and controls. The genotype distributions of the three polymorphisms of STAT3 were in Hardy–Weinberg equilibrium in control groups (*P* > 0.05).

Table 3 shows that a significant difference was observed for rs744166 between CD patients and controls both in allele and genotype distributions (*P*allele = 0.0008, and *P*genotype = 0.003). A significant decreased risk was identified for rs744166 in association with CD under the additive [odds ratio (OR) = 0.63; 95% confidence interval (CI): 0.48–0.83], dominant (OR = 0.61; 95% CI: 0.43–0.81) and recessive (OR = 0.43; 95% CI: 0.24–0.77) models.

**Table 3:** The genotype distributions and allele frequencies of the studied polymorphisms between patients and controls, and their risk prediction for CD under three genetic models of inheritance

**Polymorphism**		**CD group (%)**	**Healthy control (%)**	*** χ***^**2**^	*** P***^**†**^	**allele**	**CD group (%)**	**Healthy control (%)**	*** χ***^**2**^	*** P***^**‡**^	**CD group HWe P**^**b**^	**Healthy control HWe P**^**b**^
rs2293152	GG	58 (25.2)	78 (28.7)			G	253(55.0)	292(53.7)				
	CG	137 (59.6)	136(50)	5.16	0.08	C	207(45.0)	252(46.3)	0.18	0.67	0.21	0.93
	CC	35 (15.2)	58 (21.3)									
OR; 95% CI; P	Additive model: 094; (0.73,1.23); 0.66			Dominant model: 1.19; (0.8,1.77); 0.39					Recessive model: 0.66; (0.42,1.05); 0.08			
rs4796793	CC	111 (47.8)	112 (41.2)			C	324 (69.8)	345 (63.4)				
	CG	102 (44.0)	121 (44.5)	5.38	0.07	G	140(30.2)	199 (36.6)	4.61	0.03	0.51	0.50
	GG	19 (8.2)	39 (14.3)									
OR; 95% CI; P	Additive model: 075; (0.58,0.98); 0.03			Dominant model: 0.76; (0.57,1.09); 0.13					Recessive model: 0.53; (0.30,0.95); 0.03			
rs744166	TT	106(48)	98 (36.2)			T	309 (69.9)	323 (59.6)				
	CT	97(43.9)	127 (46.9)	11.62	0.003	C	133 (30.1)	219 (40.4)	11.28	0.0008	0.52	0.66
	CC	18 (8.1)	46 (16.9)									
OR; 95% CI; P	Additive model: 063; (0.48,0.83); <0.001			Dominant model: 0.61; (0.43,0.81); 0.008					Recessive model: 0.43; (0.24,0.77); 0.005			

*Abbreviations:HWe* Hardy-Weinberg equilibrium. P values were calculated using *χ*^2^ test 3 × 2 contingency table (†) for genotype distributions and 2 × 2 contingency table (‡) for allele distributions.OR, 95%CI and *P* values were calculated by logistic regression analysis.

As for rs4796793, a significant difference was observed between the two groups in allele but not in genotype distribution (*P*allele = 0.03 and *P*genotype = 0.07). Meanwhile, a significant decreased risk was found in association with CD under the additive (OR = 0.75; 95% CI: 0.58–0.98) and recessive (OR = 0.53; 95% CI: 0.30–0.95) models, whereas no significant association was detected under the dominant model (OR = 0.76; 95% CI: 0.57–1.09).

No significant difference was observed in the genotype and allele distributions of rs2293152 between CD patients and controls. This result also agrees under the assumptions of the additive (OR = 0.94; 95% CI: 0.73–1.23), dominant (OR = 1.19; 95% CI: 0.80–1.77) and recessive (OR = 0.66; 95% CI: 0.42–1.05) models.

## Discussion

CD is a relapsing inflammatory condition of gastrointestinal mucosal damage with characteristic extra-intestinal manifestations [22,23]. CD is widely known as an immune-related disease with genetic predisposition. Given the importance of immunity in CD, investigations on CD-susceptibility genes that involve immunity have attracted considerable attention [24,25].

The STAT3 gene is located on chromosome 17q21. Its protein product is a member of the STAT protein family that performs a dual function: signal transduction and transcription activation. STAT3 is widely expressed and a latent cytoplasmic transcription factor that relays signals from the cell membrane directly to the nucleus. STAT3 becomes activated through phosphorylation on tyrosine as a DNA-binding protein in response to a variety of stimuli and mediates the expression of a variety of genes. Thus, STAT3 possesses a key function in many biological pathways crucial to cell function, including proliferation, migration, survival and differentiation [26]. Several studies indicated that STAT3 activation plays distinctly different roles between innate and acquired immune responses in colitis, that is, activation of STAT3 in innate immune cells enhances mucosal barrier function and STAT3 activation in T-cells exacerbates colitis [11,12]. A number of studies also suggest that polymorphisms of STAT3 are associated with the susceptibility of CD or UC in some population cohorts [15-20].

We examined three polymorphisms of STAT3 in 232 CD patients and 272 normal controls of Chinese Han population. Results revealed that both the STAT3 gene alleles of rs4796793G and rs744166C reduced the risk of CD occurrence and may have a protective function in CD. To the authors' knowledge, this is the pilot study that explored the genetic susceptibility of STAT3 gene to CD in a Chinese population.

The rs744166, which was first identified as an important candidate susceptibility locus for CD in a GWAS research [14], was confirmed in a Chinese population in this study. Our results are in agreement with those previously published data in a New Zealand population [17]. They found a significant decrease in the frequency of the G allele of rs744166 in CD patients compared with controls (OR = 0.76, 95% CI = 0.61–0.95, *P* = 0.013), and G allele may be protective against CD. However, Franke et al. [15] failed to replicate the association between rs744166 and CD risk in a German population. This discrepancy may be mostly due to the heterogeneous genetic predispositions in people of different ethnicities. The genetic markers in predisposition to IBD vary across geographical and racial groups. In our previous meta-analyses, the *CD14* gene C-260 T polymorphism exhibits remarkable heterogeneity with UC across ethnic groups, which is significant in Asians but not in Caucasians [27]. However, given the relatively small samples in this study, more studies are required to reliably quantify the effect of rs744166.

rs2293152, a STAT3 variant, has been reported to be significantly associated with CD in Japanese population [16]. This variant did not show significant association between CD patient and normal control groups in this study. Sample size may be one of the major determinants because both studies (Sato’s research and our study) selected East Asia population. Sato’s study only enrolled 83 CD cases and 200 healthy controls, whereas our study included 232 CD cases and 272 normal controls. Given the larger sample size, our result seems more reliable. We could not exclude the different population results in different genetic backgrounds.

In the present study, a new candidate locus, rs4796793, was found, which was associated with CD in Chinese population. This association is not reported in other studies. Therefore, further studies should be carried out to verify this association using a large sample size from different ethnic origins and biological research.

This study has some drawbacks. First, the sample size was not very large; thus, more SNP sites for pair-loci D'/r2 value analysis and haplotype analysis on a larger number of Chinese subjects and on other ethnicities are necessary to confirm the association more clearly. Second, we only revealed limited polymorphisms of STAT3 gene associated with susceptibility to CD, and other unidentified polymorphisms, which influenced the development of CD, may still exist. Third, our results were based on unadjusted estimates. STAT3 gene polymorphisms of rs4796793 and rs744166 individually make a protective contribution against CD, but whether the polymorphisms integrated with other risk factors will change the prediction requires additional research. Thus, a more precise analysis should be conducted with individual data, which would allow for the adjustment by other co-varieties, such as age, gender, lifestyle and other genetic factors.

## Conclusion

In conclusion, this study is the first to demonstrate the single-marker association of STAT3 with CD susceptibility in the Chinese Han population. We confirmed that STAT3 rs744166 and rs4796793 polymorphisms were associated with CD occurrence and used as a predictive factor of CD in Chinese Han populations.However, the diverse genetic profiles across different ethnic groups remain unclear.

## Abbreviations

CD: Crohn’s disease; STAT3: Signal transducer and activator of transcription 3; CI: Confidence interval; UC: Ulcerative colitis; IBD: Inflammatory bowel disease; GWAS: Genome-wide association study; EDTA: Ethylene Diamine Tetraacetic Acid; PCR-SSPs: Polymerase chain reaction with sequence-specific primers; OR: Odds ratio.

## Competing interests

The authors declare that they have no competing interests.

## Authors' contributions

ZTW, BX, HXZ and J. Zhong conceived and designed the study. ZTW, HXZ, carried out the experiments and drafted the manuscript. RF, J. Zhou participated in the statistical analysis. All authors read and approved the final manuscript.
